# Phosphorylated glycosphingolipids are commonly detected in *Caenorhabditis elegans* lipidomes

**DOI:** 10.1007/s11306-024-02216-w

**Published:** 2025-02-20

**Authors:** Michael Witting, Liesa Salzer, Sven W. Meyer, Aiko Barsch

**Affiliations:** 1 Metabolomics and Proteomics Core, Helmholtz Zentrum München, Ingolstädter Landstraße 1, 85764 Neuherberg, Germany; 2 Chair of Analytical Food Chemistry, TUM School of Life Sciences, Technical University of Munich, Maximus-von-Imhof-Forum 2, 85354 Freising, Germany; 3 Research Unit Analytical BioGeoChemistry, Helmholtz Zentrum München, Ingolstädter Landstraße 1, 85764 Neuherberg, Germany; 4 Bruker Daltonics GmbH & Co. KG, Fahrenheitstraße 4, 28359 Bremen, Germany

**Keywords:** *Caenorhabditis elegans*, Sphingolipids, Trapped ion mobility, Lipidomics, Lipid identification, MassQL

## Abstract

**Introduction:**

The identification of lipids is a cornerstone of lipidomics, and due to the specific characteristics of lipids, it requires dedicated analysis workflows. Identifying novel lipids and lipid species for which no reference spectra are available is tedious and often involves a lot of manual work. Integrating high-resolution mass spectrometry with enhancements from chromatographic and ion mobility separation enables the in-depth investigation of intact lipids.

**Objectives:**

We investigated phosphorylated glycosphingolipids from the nematode *Caenorhabditis elegans*, a biomedical model organism, and aimed to identify different species from this class of lipids, which have been described in one particular publication only. We checked if these lipids can be detected in lipid extracts of *C. elegans*.

**Methods:**

We used UHPLC-UHR-TOF-MS and UHPLC-TIMS-TOF-MS in combination with dedicated data analysis to check for the presence of phosphorylated glycosphingolipids. Specifically, candidate features were identified in two datasets using Mass Spec Query Language (MassQL) to search fragmentation data. The additional use of retention time (RT) and collisional cross section (CCS) information allowed to filter false positive annotations.

**Results:**

As a result, we detected all previously described phosphorylated glycosphingolipids and novel species as well as their biosynthetic precursors in two different lipidomics datasets. MassQL significantly speeds up the process by saving time that would otherwise be spent on manual data investigations. In total over 20 sphingolipids could be described.

**Conclusion:**

MassQL allowed us to search for phosphorylated glycosphingolipids and their potential biosynthetic precursors systematically. Using orthogonal information such as RT and CCS helped filter false positive results. With the detection in two different datasets, we demonstrate that these sphingolipids are a general part of the *C. elegans* lipidome.

**Supplementary Information:**

The online version contains supplementary material available at 10.1007/s11306-024-02216-w.

## Introduction

Lipids are an essential class of biomolecules that serve as building blocks for membranes, energy storage, and even signaling molecules. The systematic analysis of lipids is referred to as lipidomics or lipid profiling. Lipids show a large structural diversity, and dependent on the lipid classes, several building blocks (fatty acyls, sphingoid bases, and headgroups) can be combined, integrating multiple lipid biosynthetic and remodeling pathways (Fahy et al., 2009; Liebisch et al., 2020; van Meer, 2005). The regulation of lipid metabolism is complex, and many model organisms have been used for its study. One of these model organisms is the small, soil-dwelling nematode *Caenorhabditis elegans* (Watts & Ristow, 2017).

**Fig. 1:**
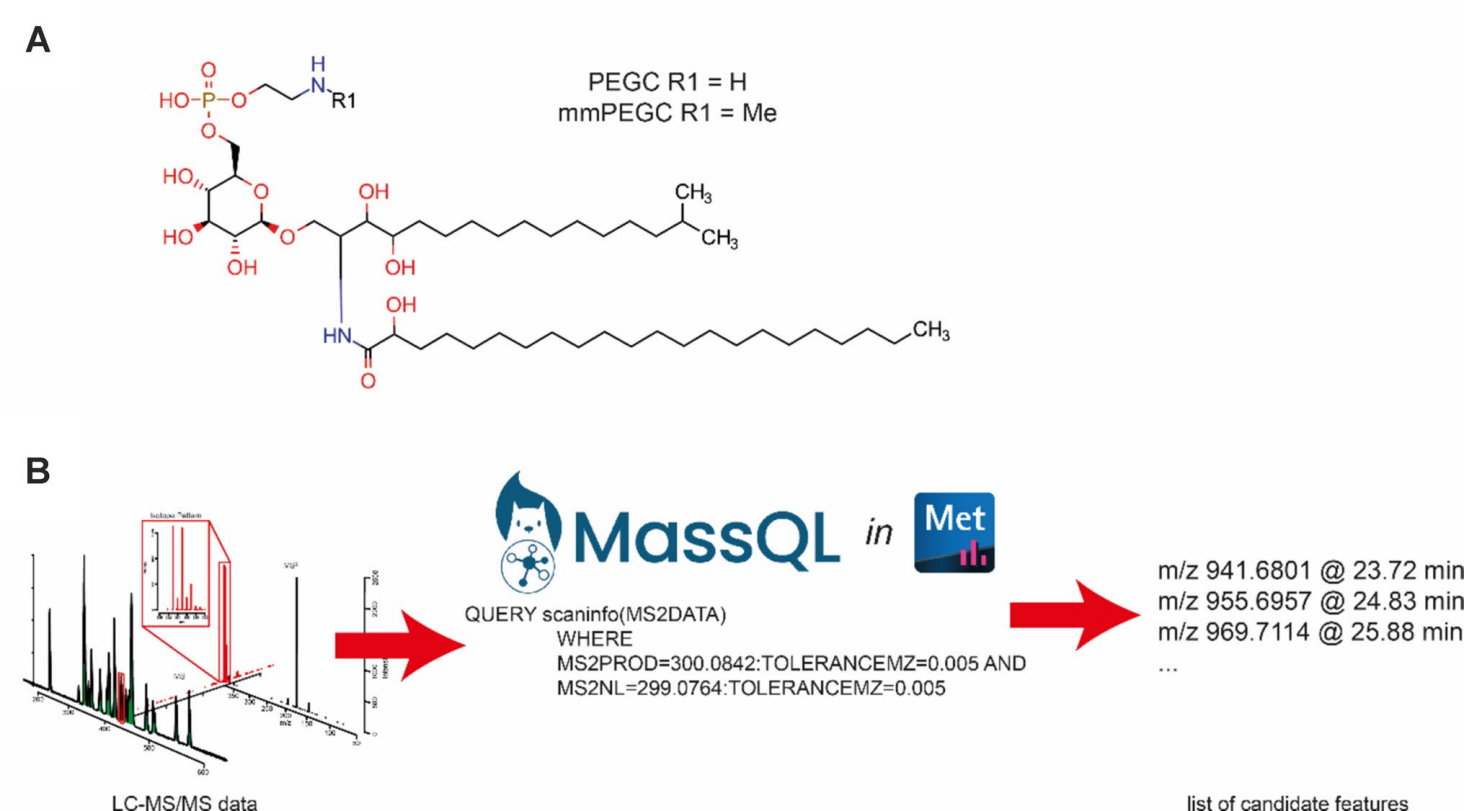
**A** Schematic structure of phosphoethanolamine glucosyl ceramides (PEGCs) and monomethyl phosphoethanolamine glucosyl ceramides found in *C. elegans*. **B** Example MassQL query used to find features potentially corresponding to mmPEGCs. Lists of features are further evaluated and filtered using RT and CCS trendlines

Sphingolipids are crucial components among the various lipid classes present in biological membranes. Together with cholesterol, they are enriched in small membrane microdomains known as lipid rafts. (Lingwood & Simons, 2010; Merris et al., 2003; Simons & Ikonen, 1997). These lipid rafts can also be found in the membranes of *C. elegans* (Rao et al., 2011; Sedensky et al., 2004). However, *C. elegans* membranes, compared to mammalian ones, contain lesser amount of cholesterol. Another striking difference is that sphingolipids in *C. elegans* contain an unusual C17 iso-branched chain sphingoid base produced from 13-methyl myristic acid and serine (Hannich et al., 2017). Furthermore, N-acyls bound in the sphingolipids usually are hydroxylated at the second carbon (Chitwood et al., 1995; Gerdt et al., 1997). It is currently unknown why *C. elegans* produce this branched chain sphingoid base, and compensation for cholesterol might be a possible reason (though it has not been proven so far). However, an essential relationship between *C. elegans* sphingolipids and cholesterol seems to exist. A novel sphingolipid class was recently described in *C. elegans*, phosphorylated glycosphingolipids. This class of sphingolipids was identified for the first time in a study investigating cholesterol deprivation (Boland et al., 2017). These novel lipids, called phosphoethanolamine glucosylceramides (PEGCs) and monomethyl phosphoethanolamine glucosyl ceramides (mmPEGCs) (structure shown in Fig. 1A), were able to rescue the larval arrest of cholesterol deprived worms. However, these lipids have only been identified in this specific study so far. They were not described in the recent in-depth analysis of the sphingolipidome of *C. elegans* (Hänel et al., 2019; Scholz et al., 2021). Missing identification of these lipids in other studies is mostly due to missing electronic reference spectra or missing structures in lipid and metabolite structure databases. Though the missing structures of the phosphorylated glycosphingolipids were curated into LipidMaps (O’Donnell et al., 2019), no electronic reference spectra have been deposited in public databases such as MassBank or GNPS (Horai et al., 2010; Wang et al., 2016), yet. These novel sphingolipids were analyzed using shotgun lipidomics without prior chromatographic separation or LC-MS analysis of isolated fractions. In our approach, we aimed to determine whether PEGCs and mmPEGCs can be more widely detected using high-resolution MS coupled with LC alone or in combination with trapped ion mobility spectrometry (TIMS). Ion mobility spectrometry (IMS) is a powerful tool for describing and identifying novel lipids. For instance, maradolipids, which are exclusively found in *C. elegans* dauer larvae (Penkov et al., 2010), have been analyzed by UHPLC-DTIM-QTOF-MS using a combination of data-independent acquisition (DIA) and ion mobility (Witting et al., 2021).

Five novel phosphorylated glycosphingolipids were reported in the work of Boland et al., two containing a phosphoethanolamine and three containing an N-methylphosphoethanolamine group. Based on the fragmentation patterns described by Boland et al. (Boland et al., 2017), we searched our recently published sphingolipidomics data (Hänel et al., 2019) to see if the described lipid species and further lipids of that same class can be detected. As a result, we detected all lipid species described by Boland et al., and additionally found one species for PEGCs and five species for mmPEGCs. To comprehensively describe this lipid class, we used UHPLC-TIMS-TOF-MS/MS to study the chromatographic and ion mobility behavior of PEGCs and mmPEGCs. Lastly, we were able to identify ceramides and hexosylceramides containing a phytosphingosine base as found in PEGCs and mmPEGCs as potential biosynthetic precursors.

## Materials and methods

### Sphingolipidome data from Hänel et al.

Data from Hänel et al. was reinvestigated. For details on lipid extraction and measurement, please refer to the original publication (Hänel et al., 2019).

### Chemicals

Methanol (MeOH), 2-propanol (iPrOH), and acetonitrile (ACN) were of LC-MS grade (Sigma-Aldrich, Taufkirchen, Germany or Biosolve Chimie, France). All other solvents and chemicals were of the highest available purity, typically analytical grade.

### Lipid extraction

*C. elegans* reference samples were obtained from the University of Georgia, Athens, Georgia, United States (Gouveia et al., 2021). The obtained material was suspended in MeOH, aliquoted into 50 µL aliquots, and lipid extraction was performed using multiple extraction methods described in detail below.

#### MeOH

An additional 400 µL of MeOH was added to the 50 µL *C. elegans* sample. The mixture was incubated at 500 rpm for one hour at 25 °C. Next, the mixture was centrifuged at 13,000 rpm for 15 min at 4 °C. The supernatant was transferred and collected, and the pellet was re-extracted with 400 µL of MeOH, followed by a second incubation at 500 rpm for 15 min at 25 °C and centrifugation at 13,000 rpm for 15 min at 4 °C. The combined supernatant was dried using a rotary vacuum concentrator until the solvent was evaporated entirely.

#### Bligh and Dyer (Bligh & Dyer, 1959)

An additional 100 µL of MeOH was added to the 50 µL *C. elegans* sample, followed by the addition of 100 µL of CHCl_3_. The mixture was incubated at 500 rpm for one hour at 25 °C. Next, 100 µL of H_2_O were added to induce phase separation. The mixture was centrifuged at 13,000 rpm for 15 min at 4 °C. The lower phase was collected in a fresh vial. The upper aqueous phase was re-extracted with 200 µL of the mixture CHCl_3_/MeOH/H_2_O (60:35:5, % v/v/v) followed by incubation at 500 rpm for 15 min at 25 °C and centrifugation at 13,000 rpm for 15 min at 4 °C. The lower organic phase was again collected and mixed with the previous CHCl_3_ phase. The CHCl_3_ phase was dried using a rotary vacuum concentrator until the solvent was evaporated entirely.

#### BUME (Löfgren et al., 2012)

300 µL of ButOH was added to the 50 µL *C. elegans* sample, followed by the addition of 400 µL Heptane/Ethyl acetate (3:1, % v/v). The mixture was incubated at 500 rpm for one hour at 25 °C. Next, 400 µL of 1% acetic acid was added to induce the phase separation; the mixture was centrifuged at 13,000 rpm for 15 min at 4 °C. The upper phase was collected in a fresh vial. The lower aqueous phase was re-extracted with 400 µL Heptane/Ethyl acetate (3:1, % v/v) followed by incubation at 500 rpm for 15 min at 25 °C and was centrifuged at 13,000 rpm for 15 min at 4 °C. The upper organic phase was collected and mixed with the previously collected organic phase. The combined organic phase was dried using a rotary vacuum concentrator until the solvent was evaporated entirely.

#### MTBE (Matyash et al., 2008)

300 µL of Methyl tert-butyl ether (MTBE) was added to the 50 µL *C. elegans* sample, and the mixture was incubated at 500 rpm for one hour at 25 °C. This was followed by the addition of 100 µL H_2_O to induce phase separation. The mixture was centrifuged at 13,000 rpm for 15 min at 4 °C. The upper phase was collected in a fresh vial. The lower aqueous phase was re-extracted with 200 µL MTBE/MeOH/ H_2_O (10:3:2.5, % v/v/v), followed by incubation at 500 rpm for 15 min at 25 °C and was centrifuged at 13,000 rpm for 15 min at 4 °C. The upper organic phase was again collected and mixed with the previously collected organic phase. The combined organic phase was dried using a rotary vacuum concentrator until the solvent was evaporated entirely.

#### Alkaline MTBE

300 µL of Methyl tert-butyl ether (MTBE) was added to the 50 µL *C. elegans* sample, and the mixture was incubated at 500 rpm for one hour at 25 °C. Afterwards, 195 µL of 1 M KOH in MeOH was added, and the mixture was incubated for two hours at 37 °C. After cooling to room temperature, 4 µL acetic acid and 376 µL of H_2_O were added to induce phase separation. The mixture was centrifuged at 13,000 rpm for 15 min at 4 °C. The upper organic phase was collected and dried using a rotary vacuum concentrator until the solvent was evaporated entirely.

### Lipidome analysis with timsTOF pro 2

Lipid analysis was performed as described previously by Witting et al. (Witting et al., 2014). Briefly, lipids were separated on a Waters Cortecs C18 column (150 mm × 2.1 mm ID, 1.6 μm particle size) (Waters, Eschborn, Germany). Elution was performed with a linear gradient. Eluent A consisted of 40% H_2_O / 60% ACN + 10 mM ammonium formate / 0.1% formic acid, and eluent B consisted of 10% ACN / 90% iPrOH + 10 mM ammonium formate / 0.1% formic acid.

Lipid extracts from *C. elegans* reference samples were analyzed using a Bruker Elute UHPLC (Bruker Daltonics GmbH & Co. KG, Bremen, Germany) coupled to a Bruker timsTOF Pro 2 (Bruker Daltonics GmbH & Co. KG, Bremen, Germany). Following gradient conditions were used: After an isocratic step with 32% B for 1.5 min, the percentage of buffer B was increased to 97% B at 21 min, held steady for 4 min, and returned to initial conditions in 0.1 min. The column was re-equilibrated for 4.9 min. The column temperature was set to 40 °C and the flow rate to 0.350 mL/min. The MS was equipped with a VIP-HESI source, and analysis was performed in positive ionization mode with the following parameters used: Sheat Gas Temperature 300 °C, Dry Gas Temperature 230 °C, Dry Gas 8.0 L/min, *m/z* 100 to 1350, Capillary voltage 4500 V, Charging voltage 2000 V, Corona 4000 nA, End plate offset − 500 V, ESI mode, Nebulizer 2.0 bar, Sheath gas flow 4.0 L/min. MS/MS was collected using DDA-PASEF, isolating single-charged molecules and fragmenting them with a collision energy of 30 eV. Trapped Ion Mobility Experiments were performed using nitrogen as carrier gas and a mobility ramp from 0.55 Vs/cm² to 1.85 Vs/cm² in 100.0 ms.

### Data Analysis

Data processing, which included *m/z* calibration, mobility calibration, peak picking, peak grouping (including de-isotoping and adduct grouping), and alignment, was performed in MetaboScape 2024b (Bruker Daltonics GmbH & Co. KG, Bremen, Germany). Both datasets discussed in Hänel et al. (2019) and the new timsTOF-based dataset acquired in this study were processed. Lipid annotation was performed using rule-based lipid annotation in MetaboScape. For the initial identification of PEGCs and mmPEGCs, a novel MassQL feature in MetaboScape 2024b was used (Fig. 1B). Further data inspection was performed in Microsoft Excel 365, including RT and CCS trendline analysis for filtering false positive candidates. Lipids belonging to the same class are expected to show an increase in RT and CCS with an increase in chain length. Plots of *m/z* vs. RT or *m/z* vs. CCS were searched for features showing a monotonous increase with increasing *m/z*. If the deviation from linear or quadratic trendlines was larger than expected, peaks were removed. All plots were generated in R 4.4.0 within RStudio (2023.06.0) using ggplot2 (3.5.1). Library spectra of PEGCs and mmPEGCs were generated from .mgf files exported from MetaboScape and multiple packages from the RforMassSpectrometry collection (Rainer et al., 2022).

## Results and discussion

### Defining fragmentation rules for PEGCs and mmPEGCs

**Fig. 2:**
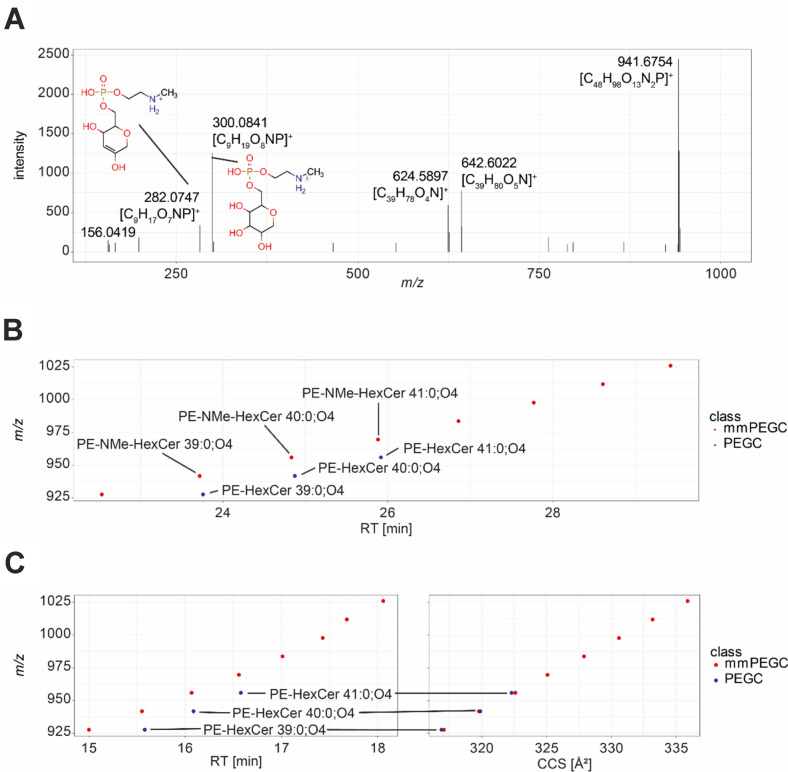
**A** Example fragmentation spectrum of PE-NMe-HexCer 39:0;O4 detected in the data from Hänel et al. (2019). Neutral loss of 299.0752 from the precursor m/z 941.6754 yields m/z 642.6022 corresponding the ceramide, which shows an additional water loss yielding m/z 624.5897. In addition, the monomethyl phosphoethanolamine glycosyl head group can be detected as m/z 300.0841 and a further water loss yielding m/z 282.0747. For high abundant species, low intensity peaks at m/z 156.0419 can be observed corresponding to the monomethyl phosphoethanolamine moiety. **B** RT trendlines for the identified PEGCs and mmPEGCs detected in the data from Hänel et al. **C** RT and CCS trendlines for the identified PEGCs and mmPEGCs from the reference *C. elegans* samples measured using a timsTOF Pro 2

Phosphoethanolamine glucosyl ceramides (PEGCs) and monomethyl phosphoethanolamine glucosyl ceramides (mmPEGCs) were identified as important, novel lipids that are essential for cholesterol mobilization in the nematode *C. elegans* (Boland et al., 2017). Two PEGCs and three mmPEGCs were initially identified using shotgun lipidomics, isolation via solid-phase extraction over silica gel and NMR. We wanted to know if more species of these two lipid classes exist and if they are only detectable under the specific conditions of the previous study or if they are generally part of the *C. elegans* lipidome. The fragmentation of PEGCs and mmPEGCs was described by Boland et al. (Boland et al., 2017). They used direct infusion mass spectrometry with an Orbitrap MS instrument. No chromatographic separation was performed. Therefore, potentially mixed fragmentation spectra of overlapping PEGCs and mmPEGCs were obtained. However, different fragments specific to each lipid class were identified. First, both classes show characteristic headgroup losses corresponding to the glucosyl moiety and the phosphoethanolamine or monomethyl phosphoethanolamine group attached. In the case of PEGCs, this neutral loss is 285.0613 (C_8_H_16_O_8_NP), and for mmPEGC the neutral loss is 299.0770 (C_9_H_18_O_8_NP). These neutral losses generate fragments of the corresponding ceramide. In addition, head group fragments can also be observed as distinct fragments at *m/z* 286.0686 ([C_8_H_17_O_8_NP]^+^) for PEGCs and at *m/z* 300.0843 ([C_9_H_19_O_8_NP]^+^) for mmPEGCs. The latter often shows an additional water loss yielding *m/z* 282.0737 ([C_9_H_17_O_7_NP]^+^) and a mono-methyl-phosphoethanolamin fragment at *m/z* 156.0420 ([C_3_H_11_O_4_NP]^+^) (Fig. 2A).

Systematic searches for MS^2^ spectra showing distinct fragments, such as the ones described above, are reoccurring tasks in metabolomics and lipidomics data analysis. For the identification of lipids in shotgun lipidomics experiments, for example, the Molecular Fragmentation Query Language (MFQL) has been proposed and has already been applied for the identification of lipids in *C. elegans* (Herzog et al., 2011; Papan et al., 2014). The Mass Spectra Query Language (MassQL) has recently been proposed as a more general way to query mass spectra and extract potentially relevant biological information (Jarmusch et al., 2022). MassQL allows for the generation of human-readable queries that can be used to search MS data for specific features, spectra, etc., showing certain properties (such fragmentation, retention, or ion mobility ranges) and has been implemented in different programming languages and software tools (including Bruker MetaboScape, which is used in this work). It is ideal to search for features associated with specific MS^2^ fragmentation.

Based on the fragmentation described for PEGCs and mmPEGCs, we generated MassQL queries to identify features potentially representing known and novel members of these two glycosphingolipid classes (Fig. 1B). These queries were applied to two datasets. Firstly, sphingolipidome profiles were obtained by Hänel et al. (2019), and secondly, a newly generated dataset of lipids detected in *C. elegans* reference samples by UHPLC-TIMS-TOF-MS/MS.

Lipids are often named using a shorthand notation, which gives information on the lipid class and its composition. To correctly name PEGCs and mmPEGCs following the established shorthand notations for lipids (Liebisch et al., 2020), we used PE-GlcCer and PE-NMe-GlcCer to denote the lipid class and notation of carbons, double bonds, and hydroxyl groups according to rules for sphingolipids, respectively. One example is PE-NME-GlcCer 16:0(15Me); O3/22:0;2OH at the level of identification according to the structures from Boland et al. (2017); this would correspond to PE-NMe-HexCer 17:0;O3/22:0;O at molecular species or PE-NMe-HexCer 39:0;O4 at species level. HexCer has been used since, for analysis at this level, (MS/MS) often, the identity of the sugar cannot be confirmed.

### Identifying PEGCs and mmPEGCs in *C. Elegans* LC-MS/MS data

We have recently performed an in-depth description of the *C. elegans* sphingolipidome using UPLC-UHR-TOF-MS/MS (Hänel et al., 2019). Extracts have been obtained from a mixed-stage sample in order to increase the number of potentially detectable sphingolipids. In this previous study, PEGCs and mmPEGCs were not investigated. We reprocessed the data using Bruker MetaboScape 2024b, which includes an implementation of MassQL as a beta version (Jarmusch et al., 2022). The following initial MassQL queries for PEGCs and mmPEGCs have been constructed:


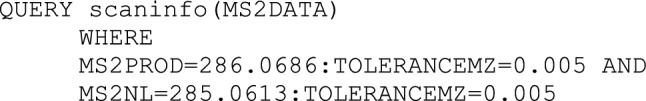



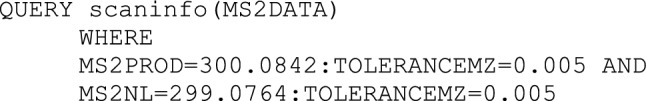


We were able to identify features showing one or the other of these fragmentation patterns. We detected two of the mmPEGCs (PE-NMe-HexCer 39:0;O4 and PE-NMe-HexCer 41:0;O4) described by Boland et al. (2017) in the dataset from Hänel et al. (2019) using MassQL for filtering together with a third feature in this dataset. The measured *m/z* matches a hypothetical mmPEGC species PE-NMe-HexCer 43:0;O4, not described so far. Since data-dependent acquisition (DDA) was used to generate MS^2^ data, further species of this lipid class might have been detected in MS^1^ and not selected for fragmentation during data acquisition. Therefore, we calculated the formula and exact mass of multiple features in silico and compared the precursor *m/z* of features associated with theoretical *m/z* values for these lipids. Since UPLC-UHR-TOF-MS/MS was used, we leveraged retention times as an additional level of information and potential filtering of false positive annotations. The three species identified by MS^2^ serve as anchor points in this scenario. In addition, we could putatively annotate PE-NMe-HexCer 40:0;O4 (also identified by Boland et al. (2017)), PE-NMe-HexCer 42:0;O4, PE-NMe-HexCer 44:0;O4 and PE-NMe-HexCer 45:0;O4 as new species of this lipid class. Plotting the *m/z* against RT, a consistent trend line was observed fitting the number of carbons in the side chains, including species confirmed by MS^2^ (see Fig. 2B). Details on the level of identification can be found in SI Table 1.

As an independent control, we checked the additional new dataset that was generated using UHPLC-TIMS-TOF-MS/MS on *C. elegans* reference samples (Gouveia et al., 2021). Similar to the samples from Hänel et al. these were extracts from mixed-stage samples. Ion mobility separation (IMS) is a valuable addition to MS, MS/MS and RT for lipid identification, e.g., to identify maradolipids in *C. elegans* (Witting et al., 2021). Besides the additional separation, which can potentially resolve isobaric and isomeric interferences, collisional cross sections (CCS) can be derived. The obtained data is largely complementary to MS and can be used for identification purposes. In contrast to RT, which represents a system’s property arising from the selected column, eluents, the analyte, and further analytical conditions, CCS are transferable between different instruments and even instrument types (e.g. TIMS, DTIMS, TWIMS) (George et al., 2024). Similar to the UPLC-UHR-TOF-MS/MS data, we used MassQL to detect putative mmPEGC species in the UHPLC-TIMS-TOF-MS/MS data. All species detected in the dataset from Hänel et al. (2019) could also be detected in this dataset with confirmation by MS^2^. Likewise, a consistent trendline across the retention time dimension was found. We also investigated the acquired CCS values in this dataset and observed a consistent trend in the mobility dimension, increasing confidence in the lipid annotations.

Next, we checked for the presence of PEGCs using the corresponding MassQL described above. No features with MS^2^ spectra fitted the query mentioned above in the dataset by Hänel et al. (2019) and only one feature matched in the UHPLC-TIMS-TOF-MS/MS dataset. Inspecting the corresponding MS^2^ spectrum in more detail, only a low abundant peak was found for the neutral loss of the headgroup. Therefore, the MassQL query was modified accordingly to potentially identify more PEGCs species:


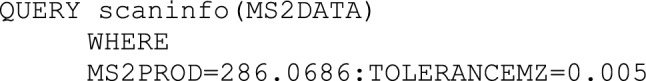


Using this modified query, several features were found in the UHPLC-TIMS-TOF-MS/MS dataset, but only three also matched the theoretical *m/z* values of PEGCs, including two species found by Boland et al. (2017), PE-HexCer 39:0;O4 and PE-HexCer 41:0;O4, and a new PE-HexCer 40:0;O4. Inspecting the RT and CCS trendlines consistent trends were found. Based on this, the data from Hänel et al. (2019) was reinvestigated. RTs of PEGCs are slightly higher compared to the corresponding isomeric mmPEGCs. Using this information and the exact *m/z* for the three lipids, features that potentially correspond to these lipids were found in the Hänel et al. (2019) dataset. However, based on matching of *m/z* values only, these annotations are highly ambiguous. Table 1 summarizes all PEGC and mmPEGC species detected in the UHPC-TIMS-MS/MS dataset.

**Table 1:** PEGC and mmPEGC species detected in the UHPLC-TIMS-TOF-MS/MS dataset

Species	Formula	m/z	RT (min)	^TIMS^CCS_N2_ (Å²)
PE-NMe-HexCer 38:0;O4	C_47_H_95_N_2_O_13_P	927.664455	15.00	317.1
PE-NMe-HexCer 39:0;O4	C_48_H_97_N_2_O_13_P	941.6801046	15.55	319.8
PE-NMe-HexCer 40:0;O4	C_49_H_99_N_2_O_13_P	955.695755	16.07	322.6
PE-NMe-HexCer 41:0;O4	C_50_H_101_N_2_O_13_P	969.7114048	16.56	325.1
PE-NMe-HexCer 42:0;O4	C_51_H_103_N_2_O_13_P	983.727055	17.01	327.9
PE-NMe-HexCer 43:0;O4	C_52_H_105_N_2_O_13_P	997.7427049	17.43	330.6
PE-NMe-HexCer 44:0;O4	C_53_H_107_N_2_O_13_P	1011.758355	17.68	333.2
PE-NMe-HexCer 45:0;O4	C_54_H_109_N_2_O_13_P	1025.774005	18.06	335.9
PE-HexCer 39:0;O4	C_47_H_95_N_2_O_13_P	927.664455	15.58	316.9
PE-HexCer 40:0;O4	C_48_H_97_N_2_O_13_P	941.6801046	16.09	319.9
PE-HexCer 41:0;O4	C_49_H_99_N_2_O_13_P	955.695755	16.58	322.3

Based on the obtained data (*m/z*, RT, MS^2^ and CCS if available), different species from both PEGCs and mmPEGCs were identified. Analysis of Boland et al. (2017) by NMR has proven that these lipids are also based on a C17 iso-branched chain sphingoid base, similar to other *C. elegans* sphingolipids. However, only in one case within the Hänel et al. (2019) dataset further evidence of a C17 sphingoid base was found: PE-NMe-HexCer 17:0;O3/24:0;O was annotated as the only species on this detailed level. So far, C17iso branched sphingoid bases are only known in *C. elegans*. Based on the assumption of a C17 sphingoid base, the N-acyls have a carbon length of 21 to 28. This matches the acyl groups known from other sphingolipid classes in the nematode (Chitwood et al., 1995; Hänel et al., 2019).

To further verify lipid species from PEGC and mmPEGC in the dataset from Hänel et al., we have performed a correlation analysis of RT values between the two datasets. A high correlation with 0.99 was found, indicating that identifications can be transferred between the datasets. Using species identified in both as “anchor” points a linear regression was used to transfer RT values. Measured RT values matched the predicted RT values with a maximum error of 1.07%.

### Ceramides and hexosylceramides based on C17-phytosphingosine are precursors for PEGCs and mmPEGCs

PEGCs and mmPEGCs show a different sphingoid base than other *C. elegans* sphingolipids. In contrast to other sphingolipids, these lipids contain a C17iso phytosphingosine base with an additional hydroxyl group at the fourth position instead of a double bond between the fourth and fifth carbon atoms. Neither ceramides nor hexosylceramides with this base have been identified by Hänel et al. or Scholz et al., performing deep analysis of the *C. elegans* sphingolipidome (Hänel et al., 2019; Scholz et al., 2021). Another study that focused on the analysis of sphingolipids was performed by Mosbech et al., but also no evidence for a C17iso phytosphingosine base was found (Mosbech et al., 2013). We searched other publications performing lipid profiling in *C. elegans* for the potential presence of these precursors of PEGCs and mmPEGCs. Lipid names were normalized to the same shorthand notations to ensure comparability. We identified four different articles that report (Anh et al., 2022; Cheng et al., 2019; Mosbech et al., 2013; Smulan et al., 2016) the presence of ceramides, hexosylceramides, or both containing potentially a phytosphingosine base. For example, Anh et al. detected one lipid species annotated as Cer 41:0;4O or Cer 17:0;3O/24:0(2OH) and one lipid species as HexCer 41:0;4O or HexCer 17:0;3O/24:0(2OH) (Anh et al., 2022).

Since no details about the used database or identification strategy were reported in most cases, we decided to reinvestigate the data from Hänel et al. to search for these sphingolipids as well. Calculating the theoretical *m/z* specific to fragments of the hypothetical phytosphingosine base (*m/z* 286.2741 ([C_17_H_36_NO_2_]^+^), *m/z* 268.2635 ([C_17_H_34_NO]^+^) and *m/z* 250.2529 ([C_17_H_32_N]^+^)) we generated the following MassQL query:


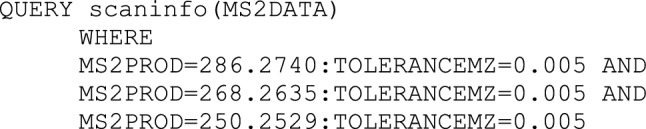


Multiple features matching this pattern were found in the mass range fitting to Cer and HexCers. First, Cers were investigated. Using exact *m/z*, fragmentation pattern, and RT trendlines, seven features were identified, including two isomers for N-acyls of length 23 and 24. One additional feature was putatively annotated based on exact *m/z* only, but it fitted well with the RT trendline as established by the MS^2^ annotated features. The two different isomers detected for the lipid species with an N-acyl of 23 and 24 carbons were baseline-separated and individually confirmed by MS^2^.

We manually inspected the spectra of putative HexCers, and additionally, the fragments from the phytosphingosine base. HexCers showed neutral losses corresponding to the hexosyl moiety and additional water losses. Therefore, a more specific MassQL query was generated and applied to search for HexCers containing a phytopshingoid base:


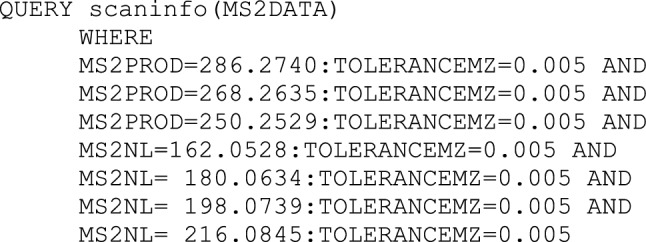


Only three features matched exactly this MassQL query, showing a high enough intensity for all fragments. More species were found with more relaxed parameters, e.g., less required fragments. Ten features were annotated as HexCers containing a phytosphingosine base, mostly on the molecular species level based on MS^2^, exact *m/z*, and RT trendlines. Only three features were annotated on the species level, but RT trends confirmed this annotation.

Sample preparation by Hänel et al. (2019) included saponification of glycerol- and glycerophospholipid, which might also lead to hydrolysis of PEGCs and mmPEGCs to HexCers and Cers. We, therefore, additionally investigated the UHPLC-TIMS-TOF-MS/MS dataset. Although this data set also included an alkaline MTBE extraction, several other extractions have been used, and the presence of Cer and HexCer in these samples confirms that these lipid classes are a native part of the *C. elegans* sphingolipidome and do not represent artifacts from sample preparation. Using the same MassQL query for general screening for phytosphingosine-based species, we found several features with a matching fragmentation spectrum; however, they were only in the mass range of Cers, not HexCers. We additionally used the rule-based lipid annotation feature within MetaboScape as an additional criterion for identification and trendlines along CCS values. 9 features were finally annotated as Cer species, with 23, 24, and 25 carbon species showing two features of potentially isomeric structures. All species were also correctly annotated by the rule-based lipid annotation. Although this feature in MetaboScape in principle, allows for the prediction of lipid CCS values, phytoceramides are currently not covered in the CCS prediction. Investigating why no HexCers were identified, we found that only the neutral loss of the hexosyl moiety could be observed due to the lower collision energy used in the UHPC-TIMS-MS/MS dataset. Accordingly, the MassQL was reduced to:


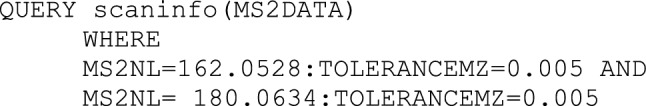


**Fig. 3:**
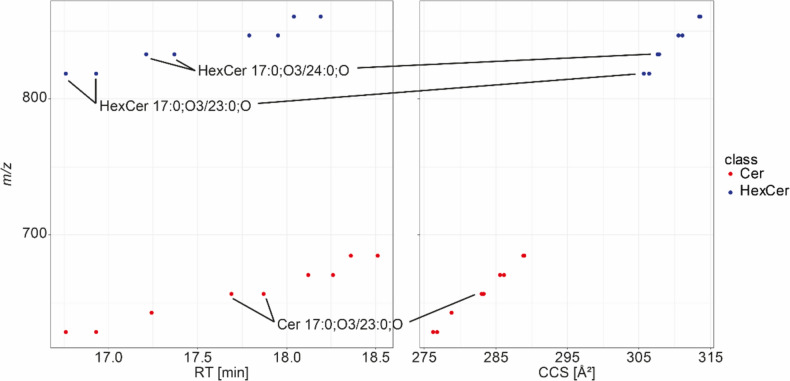
RT and CCS trendlines of HexCers and Cers containing phytosphingosine bases

Many features were filtered using this rather generic query, including a HexCer with a normal sphingoid base, indicating that this query works in principle. Again, using exact *m/z* and trendlines along RT and CCS as additional filters (Fig. 3), 11 features were annotated as HexCer containing a phytosphingosine base. These lipids were found not only in extracts from the alkaline MTBE extraction method but also in all extraction methods, suggesting they exist naturally. Based on the results obtained, Cers and HexCers with a phytosphingosine base are established as part of the *C. elegans* sphingolipidome. As precursors for PEGCs and mmPEGCs they potentially play important roles in the biology of the nematode. Similar to PEGC and mmPEGCs, RT values between the datasets were compared and high correlation was found indicating that indeed the same species have been detected between the two datasets.

## Conclusion

PEGCs and mmPEGCs were previously identified as important lipids with roles in cholesterol mobilization. Until now, these lipids have only been detected in the work of Boland et al. (2017). In their work, analysis was performed with shotgun lipidomics, leading to overlapping fragmentation spectra for isomeric species with overlapping *m/z* (e.g. PE-NMe-HexCer 40:0;O4 and PE-HexCer 41:0;O4). We argued that besides the five phosphorylated glucosylceramides (two PEGC and three mmPEGC) already identified by Boland et al. (2017), more lipids of this class is expected. Therefore, we conducted an in-depth search for additional PEGCs and mmPEGC species in two different datasets using UPLC-UHR-TOF-MS and UHPLC-TIMSTOF-MS/MS, respectively. In the first case, we reinvestigated our recently published UPLC-UHR-TOF-MS data from Hänel et al. (2019), and secondly, we performed UHPLC-TIMS-TOF-MS/MS measurements of *C. elegans* reference samples using a timsTOF Pro 2 instrument.

For a systematic search for novel lipid species, MassQL was used as a convenient way to investigate the different datasets in a systematic manner for features matching the specific fragmentation pattern of PEGCs and mmPEGCs. The additional separation dimensions of LC and TIMS helped identify species from lipid classes and Cers and HexCers based on phytosphingosine as their potential biosynthetic precursors. In addition to the two PEGCs and three mmPEGCs species identified by Boland et al. (2017), we could annotate six new phosphorylated glycosphingolipids (one PEGC and five mmPEGCs) as well as 20 Cer and HexCer species based on fragmentation pattern and trend lines along the RT and/or CCS dimension. This is the first time that these lipids (PEGCs and mmPEGCs) were analyzed by TIMS, and obtained CCS values and fragmentation spectra will serve as a reference for further investigations. Furthermore, this study serves as strong case for the combination of LC and IMS, since several of the isomeric/isobaric pairs of lipids detected could not be resolved by IMS alone. Furthermore, the correlation of RT values between the two datasets showed high values of 0.99 for all investigated lipid classes, indicating that, indeed, the same species were identified in both datasets.

Our results indicate that PEGCs and mmPEGCs can be detected in *C. elegans* using multiple lipid extraction methods. This suggests that these lipids might as well be detected in the *C. elegans* lipidome beyond the study by Boland et al. (2017) and this study. We did a retrospective analysis of several other datasets created by us during the last years and were able to detect PEGCs and mmPEGCs in multiple datasets, e.g., Rackles et al. and Haeussler et al. (data not shown) (Haeussler et al., 2020; Rackles et al., 2021). Using MassQL for mmPEGC within the MetaboScape software, we found in both datasets mmPEGC(q39:0) and mmPEGC(q41:0). The same was true for several other unpublished datasets. Unfortunately, not many further public *C. elegans* lipidomics datasets were available to check for the presence of PEGCs or mmPEGCs beyond our own data. We investigated a dataset uploaded to Metabolomics Workbench data (Sud et al., 2016) that contained lipid profiling data. Exact masses matching the theoretical masses of PEGCs and mmPEGCs could be annotated, but due to missing MS^2^ information not further confirmed. Based on the results, PEGCs and mmPEGCs are part of the lipidome of *C. elegans*, and we suggest they should be included in future investigations into sphingolipids.

## Electronic supplementary material

Below is the link to the electronic supplementary material.

Supplementary file1 (DOCX 27 kb)

Supplementary file2 (MB 28 kb)

Supplementary file3 (MB 57 kb)

## Data Availability

Data from Hänel et al. is available from MetaboLights “MTBLS963: The Sphingolipidome of the model organism Caenorhabditis elegans”. Generated reference spectra are available in the SI of this article in MassBank record format.
