# Supplementary material for: Phosphorylated glycosphingolipids are commonly detected in Caenorhabditis elegans lipidomes
Source: Metabolomics. 2025 Feb 20;21(2):29. doi: 10.1007/s11306-024-02216-w (PMC11842410; doi:10.1007/s11306-024-02216-w)
Supplement: Supplementary file 1 — Supplementary file1 (DOCX 27 kb) [file 11306_2024_2216_MOESM1_ESM.docx]

**Supporting information**

Reference spectra from identified sphingolipids are available as SI Data 1 (Spectra from Hänel *et al.*) and SI data 2 (Spectra from timsTOF datasets). Data is in MassBank record format.

SI Table 1: PEGCs and mmPEGCs detected in the dataset by Hänel et al. Lipids were annotated based on different evidence levels: exact m/z means the measured m/z is fitting the theoretical m/z within 0.005 Da, MS^2^ means, that a fitting MS2 pattern has been found either manually or by MassQL, RT trendlines mean that this features follows the trendline for the specific lipid clas in an m/z versus RT plot, CCS trendline means that this feature follows the trendline for the specific lipid class in an m/z vs CCS plot

| **Identification** | **Evidence** | ***m/z*** | **RT (min)** | **^TIMS^CCS_N2_ (Å²)** |
| --- | --- | --- | --- | --- |
| PE-HexCer 39:0;O4  C_47_H_95_N_2_O_13_P  *m/z* 927.6645 [M+H]^+^ | - exact *m/z* | 927.6615  (-3.20 ppm) | 23.76 | --- |
| PE-HexCer 40:0;O4  C_48_H_97_N_2_O_13_P  *m/z* 941.6801 [M+H]^+^ | - exact *m/z* | 9416771  (-3.20 ppm) | 24.87 | --- |
| PE-HexCer 41:0;O4  C_49_H_99_N_2_O_13_P  *m/z* 955.6958 [M+H]^+^ | - exact *m/z* | 955.6937  (-2.19 ppm) | 25.92 | --- |
| PE-NMe-HexCer 38:0;O4  C_47_H_95_N_2_O_13_P  *m/z* 927.6645 [M+H]^+^ | - exact *m/z*  - RT trendline | 927.6624  (-2.20 ppm) | 22.53 | --- |
| PE-NMe-HexCer 39:0;O4  C_48_H_97_N_2_O_13_P  *m/z* 941.6801 [M+H]^+^ | - MS^2^ (MassQL)  - exact *m/z*  - RT trendline | 941.6786  (-1.63 ppm) | 23.72 | --- |
| PE-NMe-HexCer 40:0;O4  C_49_H_99_N_2_O_13_P  *m/z* 955.6958 [M+H]^+^ | - exact *m/z*  - RT trendline | 955.6935  (-2.38 ppm) | 24.83 | --- |
| PE-NMe-HexCer 17:0;O3/24:0;O  C_50_H_101_N_2_O_13_P  *m/z* 969.7114 [M+H]^+^ | - MS^2^ (MassQL)  - exact *m/z*  - RT trendline | 969.7103  (-1.10 ppm) | 25.88 | ---- |
| PE-NMe-HexCer 42:0;O4  C_51_H_103_N_2_O_13_P  *m/z* 983.7271 [M+H]^+^ | - exact *m/z*  - RT trendline | 983.7255  (-1.62 ppm) | 26.86 | ---- |
| PE-NMe-HexCer 43:0;O4  C_52_H_105_N_2_O_13_P  *m/z* 997.7427 [M+H]^+^ | - MS^2^ (MassQL)  - exact *m/z*  - RT trendline | 997.7410  (-1.72 ppm) | 27.77 | --- |
| PE-NMe-HexCer 44:0;O4  C_53_H_107_N_2_O_13_P  *m/z* 1011.7584 [M+H]^+^ | - exact *m/z*  - RT trendline | 1011.7563  (-2.23 ppm) | 28.61 | ---- |
| PE-NMe-HexCer 45:0;O4  C_54_H_109_N_2_O_13_P  *m/z* 1025.7740 [M+H]^+^ | - exact *m/z*  - RT trendline | 1025.7717  (-2.07 ppm) | 29.43 | ---- |

SI Table 2: PEGCs and mmPEGCs detected in the dataset by UHPLC-TIMSTOF-MS/MS. Lipids were annotated based on different evidence levels: exact m/z means the measured m/z is fitting the theoretical m/z within 0.005 Da, MS^2^ means, that a fitting MS2 pattern has been found either manually or by MassQL, RT trendlines mean that this features follows the trendline for the specific lipid clas in an m/z versus RT plot, CCS trendline means that this feature follows the trendline for the specific lipid class in an m/z vs CCS plot

| **Identification** | **Evidence** | ***m/z*** | **RT (min)** | **^TIMS^CCS_N2_ (Å²)** |
| --- | --- | --- | --- | --- |
| PE-HexCer 39:0;O4  C_47_H_95_N_2_O_13_P  *m/z* 927.6645 [M+H]^+^ | - MS^2^ (MassQL)  - exact *m/z*  - RT trendline  - CCS trendline | 927.6645  (0.03 ppm) | 15.58 | 316.9 |
| PE-HexCer 40:0;O4  C_48_H_97_N_2_O_13_P  *m/z* 941.6801 [M+H]^+^ | - MS^2^ (MassQL)  - exact *m/z*  - RT trendline  - CCS trendline | 941.6811  (1.01 ppm) | 16.09 | 319.9 |
| PE-HexCer 41:0;O4  C_49_H_99_N_2_O_13_P  *m/z* 955.6958 [M+H]^+^ | - MS^2^ (MassQL)  - exact *m/z*  - RT trendline  - CCS trendline | 955.6970  (1.29 ppm) | 16.58 | 322.3 |
| PE-NMe-HexCer 38:0;O4  C_47_H_95_N_2_O_13_P  *m/z* 927.6645 [M+H]^+^ | - MS^2^ (MassQL)  - exact *m/z*  - RT trendline  - CCS trendline | 927.6647  (0.29 ppm) | 15.00 | 317.1 |
| PE-NMe-HexCer 39:0;O4  C_48_H_97_N_2_O_13_P  *m/z* 941.6801 [M+H]^+^ | - MS^2^ (MassQL)  - exact *m/z*  - RT trendline  - CCS trendline | 941.6804  (0.35 ppm) | 15.55 | 319.8 |
| PE-NMe-HexCer 40:0;O4  C_49_H_99_N_2_O_13_P  *m/z* 955.6958 [M+H]^+^ | - MS^2^ (MassQL)  - exact *m/z*  - RT trendline  - CCS trendline | 955.6961  (0.40 ppm) | 16.07 | 322.6 |
| PE-NMe-HexCer 41:0;O4  C_50_H_101_N_2_O_13_P  *m/z* 969.7114 [M+H]^+^ | - MS^2^ (MassQL)  - exact *m/z*  - RT trendline  - CCS trendline | 969.7116  (0.20 ppm) | 16.56 | 325.1 |
| PE-NMe-HexCer 42:0;O4  C_51_H_103_N_2_O_13_P  *m/z* 983.7271 [M+H]^+^ | - MS^2^ (MassQL)  - exact *m/z*  - RT trendline  - CCS trendline | 983.7273  (0.29 ppm) | 17.01 | 327.9 |
| PE-NMe-HexCer 43:0;O4  C_52_H_105_N_2_O_13_P  *m/z* 997.7427 [M+H]^+^ | - MS^2^ (MassQL)  - exact *m/z*  - RT trendline  - CCS trendline | 997.7427  (0.03 ppm) | 17.43 | 330.6 |
| PE-NMe-HexCer 44:0;O4  C_53_H_107_N_2_O_13_P  *m/z* 1011.7584 [M+H]^+^ | - MS^2^ (MassQL)  - exact *m/z*  - RT trendline  - CCS trendline | 1011.7584  (0.04 ppm) | 17.68 | 333.2 |
| PE-NMe-HexCer 45:0;O4  C_54_H_109_N_2_O_13_P  *m/z* 1025.7740 [M+H]^+^ | - MS^2^ (MassQL)  - exact *m/z*  - RT trendline  - CCS trendline | 1025.7753  (1.29 ppm) | 18.06 | 335.9 |

SI Table 3: Cers with phytosphingosine base in Hänel et al. dataset Lipids were annotated based on different evidence levels: exact m/z means the measured m/z is fitting the theoretical m/z within 0.005 Da, MS^2^ means, that a fitting MS2 pattern has been found either manually or by MassQL, RT trendlines mean that this features follows the trendline for the specific lipid clas in an m/z versus RT plot, CCS trendline means that this feature follows the trendline for the specific lipid class in an m/z vs CCS plot

| **Identification** | **Evidence** | ***m/z*** | **RT (min)** | **^TIMS^CCS_N2_ (Å²)** |
| --- | --- | --- | --- | --- |
| Cer 17:0;O3/21:0;O  C_38_H_77_NO_5_  *m/z* 628.5875 [M+H]^+^ | - MS^2^ (MassQL)  - exact *m/z*  - RT trendline | 628.5870  (-0.78 ppm) | 26.66 | --- |
| Cer 38:0;O4  C_38_H_77_NO_5_  *m/z* 628.5875 [M+H]^+^ | - exact *m/z*  - RT trendline | 628.5860  (-2.32 ppm) | 27 | --- |
| Cer 17:0;O3;22:0;O  C_39_H_79_NO_5_  *m/z* 643.6013 [M+H]^+^ | - MS^2^  - exact *m/z*  - RT trendline | 642.6021  (-1.56 ppm) | 27.68 | --- |
| Cer 17:0;O3/23:0;O  C_40_H_81_NO_5_  *m/z* 656.6188 [M+H]^+^ | - MS^2^ (MassQL)  - exact *m/z*  - RT trendline | 656.6179  (-1.24 ppm) | 28.3 | --- |
| Cer 17:0;O3/23:0;O  C_40_H_81_NO_5_  *m/z* 656.6188 [M+H]^+^ | - MS^2^ (MassQL)  - exact *m/z*  - RT trendline | 656.6186  (-0.23 ppm) | 28.64 | --- |
| Cer 17:0;O3/24:0;O  C_41_H_83_NO_5_  *m/z* 670.6344 [M+H]^+^ | - MS^2^ (MassQL)  - exact *m/z*  - RT trendline | 670.6341  (-0.40 ppm) | 29.2 | --- |
| Cer 17:0;O3/24:0;O  C_41_H_83_NO_5_  *m/z* 670.6344 [M+H]^+^ | - MS^2^ (MassQL)  - exact *m/z*  - RT trendline | 670.6339  (-0.82 ppm) | 29.52 | --- |

SI Table 4: Ceramides with phytosphingosine base in UHPLC-TIMSTOF-MS/MS dataset Lipids were annotated based on different evidence levels: exact m/z means the measured m/z is fitting the theoretical m/z within 0.005 Da, MS^2^ means, that a fitting MS2 pattern has been found either manually or by MassQL, RT trendlines mean that this features follows the trendline for the specific lipid clas in an m/z versus RT plot, CCS trendline means that this feature follows the trendline for the specific lipid class in an m/z vs CCS plot

| **Identification** | **Evidence** | ***m/z*** | **RT (min)** | **^TIMS^CCS_N2_ (Å²)** |
| --- | --- | --- | --- | --- |
| Cer 17:0;O3/21:0;O  C_38_H_77_NO_5_  *m/z* 628.5875 [M+H]^+^ | - MS^2^ (MassQL)  - exact *m/z*  - RT trendline  - CCS trendline | 628.5875  (0.06 ppm) | 16.76 | 276.8 |
| Cer 38:0;O4  C_38_H_77_NO_5_  *m/z* 628.5875 [M+H]^+^ | - exact *m/z*  - RT trendline  - CCS trendline | 628.5872  (-0.35 ppm) | 16.93 | 276.2 |
| Cer 17:0;O3/22:0;O  C_39_H_79_NO_5_  *m/z* 643.6013 [M+H]^+^ | - MS^2^ (MassQL)  - exact *m/z*  - RT trendline  - CCS trendline | 642.6031  (-0.06 ppm) | 17.24 | 278.8 |
| Cer 17:0;O3/23:0;O  C_40_H_81_NO_5_  *m/z* 656.6188 [M+H]^+^ | - MS^2^ (MassQL)  - exact *m/z*  - RT trendline  - CCS trendline | 656.6188  (0.06 ppm) | 17.69 | 283 |
| Cer 40:0;O4  C_40_H_81_NO_5_  *m/z* 656.6188 [M+H]^+^ | - exact *m/z*  - RT trendline  - CCS trendline | 656.6193  (0.81 ppm) | 17.87 | 283.3 |
| Cer 17:0;O3/24:0;O  C_41_H_83_NO_5_  *m/z* 670.6344 [M+H]^+^ | - MS^2^ (MassQL)  - exact *m/z*  - RT trendline  - CCS trendline | 670.6348  (0.57 ppm) | 18.12 | 286.1 |
| Cer 17:0;O3/24:0;O  C_41_H_83_NO_5_  *m/z* 670.6344 [M+H]^+^ | - MS^2^ (MassQL)  - exact *m/z*  - RT trendline  - CCS trendline | 670.6322  (-3.36 ppm) | 18.26 | 285.6 |
| Cer 17:0;O3/25:0;O  C_42_H_85_NO_5_  *m/z* 684.6500 [M+H]^+^ | - MS^2^ (MassQL)  - exact *m/z*  - RT trendline  - CCS trendline | 684.6498  (-0.38 ppm) | 18.36 | 289 |
| Cer 17:0;O3/25:0;O  C_42_H_85_NO_5_  *m/z* 684.6500 [M+H]^+^ | - MS^2^ (MassQL)  - exact *m/z*  - RT trendline  - CCS trendline | 684.6499  (-0.16 ppm) | 18.51 | 288.8 |

SI Table 5: HexCers with phytosphingosine base from Hänel et al. Lipids were annotated based on different evidence levels: exact m/z means the measured m/z is fitting the theoretical m/z within 0.005 Da, MS^2^ means, that a fitting MS2 pattern has been found either manually or by MassQL, RT trendlines mean that this features follows the trendline for the specific lipid clas in an m/z versus RT plot, CCS trendline means that this feature follows the trendline for the specific lipid class in an m/z vs CCS plot

| **Identification** | **Evidence** | ***m/z*** | **RT (min)** | **^TIMS^CCS_N2_ (Å²)** |
| --- | --- | --- | --- | --- |
| HexCer 17:0;O3/22:0;O  C_45_H_89_NO_10_  *m/z* 804.6559 [M+H]^+^ | - MS^2^ (MassQL)  - exact *m/z*  - RT trendline | 804.656  (-0.42 ppm) | 26.2 | --- |
| HexCer 40:0;O4  C_46_H_91_NO_10_  *m/z* 818.6716 [M+H]^+^ | - exact *m/z*  - RT trendline | 818.6701  (-1.86 ppm) | 26.87 | --- |
| HexCer 17:0;O4/23:0;O  C_46_H_91_NO_10_  *m/z* 818.6716 [M+H]^+^ | - MS^2^  - exact *m/z*  - RT trendline | 818.6705  (-1.26 ppm) | 27.21 | --- |
| HexCer 17:0;O4/24:0;O  C_47_H_93_NO_10_  *m/z* 832.6872 [M+H]^+^ | - MS^2^  - exact *m/z*  - RT trendline | 832.6858  (-1.73 ppm) | 27.83 | --- |
| HexCer 17:0;O4/24:0;O  C_47_H_93_NO_10_  *m/z* 832.6872 [M+H]^+^ | - MS^2^ (MassQL)  - exact *m/z*  - RT trendline | 832.6865  (-0.93 ppm) | 28.16 | --- |
| HexCer 17:0;O4/25:0;O  C_48_H_95_NO_10_  *m/z* 846.7029 [M+H]^+^ | - MS^2^  - exact *m/z*  - RT trendline | 846.7015  (-1.66 ppm) | 28.73 | --- |
| HexCer 17:0;O4/25:0;O  C_48_H_95_NO_10_  *m/z* 846.7029 [M+H]^+^ | - MS^2^ (MassQL)  - exact *m/z*  - RT trendline | 846.7017  (-1.34 ppm) | 29.04 | --- |
| HexCer 43:0;O4  C_49_H_97_NO_10_  *m/z* 860.7185 [M+H]^+^ | - exact *m/z*  - RT trendline | 860.7173  (-1.47 ppm) | 29.59 | --- |
| HexCer 17:0;O3/26:0;O  C_49_H_97_NO_10_  *m/z* 860.7185 [M+H]^+^ | - MS^2^  - exact *m/z*  - RT trendline | 860.7167  (-2.16 ppm) | 29.87 | --- |

SI Table 6: HexCers with phytosphingosine base from timsTOF datasets. Lipids were annotated based on different evidence levels: exact m/z means the measured m/z is fitting the theoretical m/z within 0.005 Da, MS^2^ means, that a fitting MS2 pattern has been found either manually or by MassQL, RT trendlines mean that this features follows the trendline for the specific lipid clas in an m/z versus RT plot, CCS trendline means that this feature follows the trendline for the specific lipid class in an m/z vs CCS plot

| **Identification** | **Evidence** | ***m/z*** | **RT (min)** | **^TIMS^CCS_N2_ (Å²)** |
| --- | --- | --- | --- | --- |
| HexCer 38:0;O4  C_44_H_87_NO_10_  *m/z* 790.6403 [M+H]^+^ | - exact *m/z*  - RT trendline  - CCS trendline | 790.6413  (1.25 ppm) | 15.94 | 299.9 |
| HexCer 17:0;O3/22:0;O  C_45_H_89_NO_10_  *m/z* 804.6559 [M+H]^+^ | - MS^2^ (MassQL)  - exact *m/z*  - RT trendline  - CCS trendline | 804.6561  (0.26 ppm) | 16.45 | 301.5 |
| HexCer 17:0;O4/23:0;O  C_46_H_91_NO_10_  *m/z* 818.6716 [M+H]^+^ | - MS^2^ (MassQL)  - exact *m/z*  - RT trendline  - CCS trendline | 818.6728  (1.50 ppm) | 16.76 | 306.4 |
| HexCer 17:0;O4/23:0;O  C_46_H_91_NO_10_  *m/z* 818.6716 [M+H]^+^ | - MS^2^ (MassQL)  - exact *m/z*  - RT trendline  - CCS trendline | 818.6716  (0.07 ppm) | 16.93 | 305.7 |
| HexCer 17:0;O4/24:0;O  C_47_H_93_NO_10_  *m/z* 832.6872 [M+H]^+^ | - MS^2^ (MassQL)  - exact *m/z*  - RT trendline  - CCS trendline | 832.6875  (0.33 ppm) | 17.21 | 307.6 |
| HexCer 41:0;O4  C_47_H_93_NO_10_  *m/z* 832.6872 [M+H]^+^ | - MS^2^  - exact *m/z*  - RT trendline  - CCS trendline | 832.6874  (0.17 ppm) | 17.37 | 307.8 |
| HexCer 17:0;O4/25:0;O  C_48_H_95_NO_10_  *m/z* 846.7029 [M+H]^+^ | - MS^2^  - exact *m/z*  - RT trendline  - CCS trendline | 846.7030  (0.18 ppm) | 17.79 | 311.1 |
| HexCer 42:0;O4  C_48_H_95_NO_10_  *m/z* 846.7029 [M+H]^+^ | - MS^2^  - exact *m/z*  - RT trendline  - CCS trendline | 846.7037  (0.99 ppm) | 17.95 | 310.5 |
| HexCer 43:0;O4  C_49_H_97_NO_10_  *m/z* 860.7185 [M+H]^+^ | - MS^2^  - exact *m/z*  - RT trendline  - CCS trendline | 860.7199  (1.61 ppm) | 18.04 | 313.4 |
| HexCer 43:0;O4  C_49_H_97_NO_10_  *m/z* 860.7185 [M+H]^+^ | - MS^2^  - exact *m/z*  - RT trendline  - CCS trendline | 860.7197  (1.32 ppm) | 18.19 | 313.6 |
